# Turning the tide on sex and the microbiota in aquatic animals

**DOI:** 10.1007/s10750-022-04862-4

**Published:** 2022-05-05

**Authors:** Kieran A. Bates, Chelsea Higgins, Maurine Neiman, Kayla C. King

**Affiliations:** 1 Department of Zoology, University of Oxford, Oxford, OX1 3SZ UK; 2 Department of Biology, University of Iowa, Iowa City, IW 52245 USA; 3 Department of Gender, Women’s, and Sexuality Studies, University of Iowa, Iowa City, IW 52245 USA

**Keywords:** Microbiome, Aquatic animals, Sex

## Abstract

Sex-based differences in animal microbiota are increasingly recognized as of biological importance. While most animal biomass is found in aquatic ecosystems and many water-dwelling species are of high economic and ecological value, biological sex is rarely included as an explanatory variable in studies of the aquatic animal microbiota. In this opinion piece, we argue for greater consideration of host sex in studying the microbiota of aquatic animals, emphasizing the many advancements that this information could provide in the life sciences, from the evolution of sex to aquaculture.

## Introduction

Among sexually reproducing animals, males and females typically exhibit distinct physiological and morphological traits driven at least in part by differences in sex-specific selection pressures. These sex-specific asymmetries can in turn have evolutionarily important consequences, for example by driving speciation (Darwin, 1871; West-Eberhard, 1983; Gavrilets, 2000; Panhuis et al., 2001; Servedio & Boughman, 2017). In recent years, sexual differences in host biology have been shown to be associated with differences in resident microbial communities (the microbiota) across animal hosts, including humans (Mueller et al., 2006; Markle et al., 2013; Yurkovetskiy et al., 2013; Bolnick et al., 2014; de la Cuesta-Zuluaga et al., 2019; Ma & Li, 2019; Sinha et al., 2019; Janiak et al., 2021). Animal-associated microbiota also play vital roles in host health (Ottman et al., 2012), impacting metabolism (Fan & Pedersen, 2021), behaviour (Johnson & Foster, 2018), development (Shin et al., 2011), and response to infection (Hooper et al., 2012; Stevens et al., 2021). In many instances, these processes are moderated by host sex (Jašarević et al., 2016; Baars et al., 2018; Elderman et al., 2018; Weger et al., 2019).

**Fig. 1:**
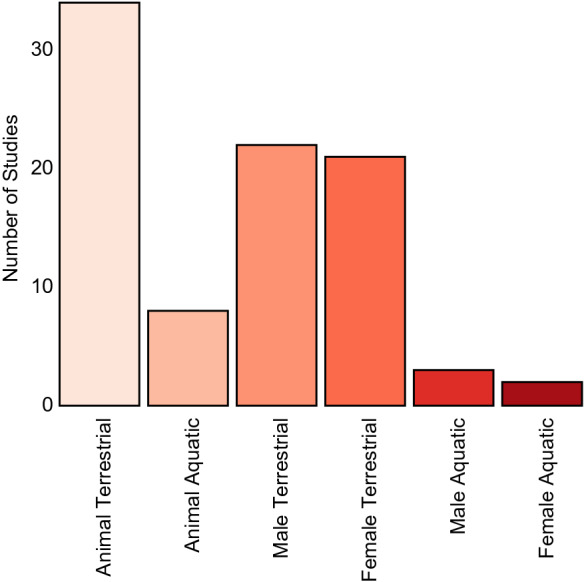
Bar plot of number of Earth Microbiome Project Database (The Earth Microbiome Project Consortium et al., 2017) studies based on terrestrial or aquatic environment and host sex in animals. Sample metadata were searched using the tool rediom (McDonald et al., 2019) with the terms “animal & terrestrial/aquatic & male/female” within the context “Deblur-Illumina-16S-V4-90nt-99d1d8” which was selected based on having the highest number of samples

Although the majority of animal biomass is found in the oceans (Bar-On et al., 2018) and despite the ecological/economic importance of aquatic ecosystems (Geist, 2011; Food and Agriculture Organisation of the United Nations [FAO], 2016), the microbiota of aquatic animals is often overlooked compared to terrestrial taxa (Fig. 1). This is an important knowledge gap in light of the fact that aquatic and terrestrial environments differ in ways likely to impact host biology and microbial ecology (Grummer et al., 2019). Water is at least 40 times more viscous and ~ 800 times denser than air. Water also has substantially higher thermal conductivity and capacity. Oxygen solubility exhibits an inverse relationship with water temperature, a property that likely drives adaptation in aquatic animals (Chen et al., 2018b; Sandoval‐Castillo et al., 2018) and influences microbial communities (Spietz et al., 2015; Sunagawa et al., 2015; Ullah Khan et al., 2021). The variable flow/current of water in aquatic environments further impacts microbial locomotion and ecology (Rusconi et al., 2014) as well as shapes host microbial communities (Lee et al., 2017). Finally, aquatic ecosystems typically have high connectivity, meaning that organisms often encounter a range of habitats over their lifetime, each with their own unique stressors (Grummer et al., 2019). When examining fundamental biological questions, such as the association between the microbiota and host sex, it is therefore essential that we include the aquatic environment. Doing so will help us gain a holistic view of the biological processes underpinning the ecology and evolution of animal life.

In this opinion piece, we discuss how host sex shapes the microbiota of aquatic animals. We demonstrate how the diversity of sexual systems exhibited by aquatic animals provide powerful models for examining how sex might structure host microbiotas and vice versa. We also discuss the strengths that aquatic animal systems offer when it comes to studying the intersection between the microbiota and the ecology/evolution of animals. Finally, we highlight how consideration of sex-specific microbiotas may benefit species conservation and aquaculture.

## Sexual differences in the microbiota of aquatic animals

Sexual signatures in the microbial communities of aquatic animals have been found across diverse host taxa including invertebrates, fish, and marine mammals (Table 1). Although very few studies have investigated how host sex influences the microbiota of aquatic invertebrates, sexual differences have been reported in coral (Wessels et al., 2017), intertidal crustaceans (Wenzel et al., 2018; Clarke et al., 2019), cephalopods (Iehata et al., 2015), and gastropods (Takacs-Vesbach et al., 2016). In a study of intertidal isopods (*Jaera albifrons* Leach, 1814), sex was attributed to 14% of variation in microbial beta diversity, with higher alpha diversity also reported in males (Wenzel et al., 2018). The authors hypothesize that these sexual differences in microbial communities could be linked to sexual size dimorphism (Veuille, 1980) or to sex-specific differences in habitat selection (Merilaita & Jormalainen, 1997). Microbial community functional differences have also been observed based on host sex. For example, bacterial community structure and nutritional enzyme activity in the digestive tract of the Chilean octopus (*Octopus mimus* Gould,1852) were shown to differ between males and females, with males also showing higher bacterial alpha diversity (Iehata et al., 2015).

**Table 1:** Examples of observed sexual differences in the microbiota of selected aquatic animals

Phylum	Species	Sexual or Asexual	Microbial associations as a function of sex
Mollusca	New Zealand Mud Snail; (*Potamopyrgus antipodarum* (Gray, 1843))	Sexual or asexual	Sexual and asexual animals had a mean beta dissimilarity of 90% (Takacs-Vesbach et al., 2016)
Mollusca	Chilean Octopus (*Octopus mimus* Gould, 1852)	Sexual	Digestive tract bacterial community structure and nutritional enzyme activity differed between males and females, with males showing higher alpha diversity (Iehata et al., 2015)
Cnidaria	Octocoral (*Lobophytum pauciflorum* (Ehrenberg, 1834))	Sexual	Some suggestion that males and females differed in community structure: *Spirochaetes*- and *Rhodobacteraceae*-related sequences more abundant in males than in female corals (1.4 × and 4x, respectively) (Wessels et al., 2017)
Arthropoda	Intertidal isopod (*Jaera albifrons* Leach, 1814)	Sexual	14.1% of variation in bacterial beta diversity could be attributed to host sex (Wenzel et al., 2018)
Vertebrata	Three-spined stickleback; (*Gasterosteus aculeatus* Linnaeus, 1758)	Sexual	Among-individual diet variation was correlated with individual differences in gut microbiota in a sex-dependent fashion (Bolnick et al., 2014)
Vertebrata	Eurasian perch; (*Perca fluviatilis* Linnaeus, 1758)	Sexual	Among-individual diet variation was correlated with individual differences in gut microbiota in a sex-dependent fashion (Bolnick et al., 2014). Gut microbial community reacts to predation stress and food rationing in sex-dependent manner (Zha et al., 2018)
Vertebrata	Zebrafish (*Danio rerio* (Hamilton, 1822))	Sexual	Exposure to titanium dioxide in combination with bisphenol A shifted gut microbiota and host physiology differently between males and females (Chen et al., 2018a)
Vertebrata	Fathead minnow (*Pimephales promelas* Rafinesque, 1820)	Sexual	Females exhibited higher gut bacterial Shannon diversity, differences in beta diversity, taxon abundance, and predicted functional pathways relative to males. Low-dose exposure of the polycyclic hydrocarbon BaP (PAH benzo[a]pyrene), disturbed the gut microbiota structure of females, but not males (DeBofsky et al., 2020)
Vertebrata	Elephant Seal (*Mirounga leonina* (Linnaeus, 1758))	Sexual	Significant difference in gut microbial community of males and females (Nelson et al., 2013b)
Vertebrata	Beluga whales (*Delphinapterus leucas* (Pallas, 1776))	Sexual	Significant differences in epidermal microbiota of males and females (Van Cise et al., 2020)

Among marine mammals, which differ in their gut microbiota compared to terrestrial relatives (Nelson et al., 2013a), the majority of studies indicate that the microbiota is not strongly influenced by host sex. Studies in leopard seals (*Hydrurga leptonyx* (Blainville, 1820)) (Nelson et al., 2013b), dugongs (*Dugong dugon* (Müller, 1776)) (Eigeland, 2012), manatees (*Trichechus manatus* Linnaeus, 1758) (Merson et al., 2014), bottle nose dolphins (*Tursiops truncatus* (Montagu, 1821)) (Bik et al., 2016), and kogiid whales (*Kogia sima* (Owen 1866) & *Kogia breviceps* (de Blainville, 1838)) (Erwin et al., 2017; Denison et al., 2020) have all not found a significant association between host sex and gut microbiota. By contrast, elephant seals (*Mirounga leonina* (Linnaeus, 1758)) do exhibit pronounced differences in the microbial communities of males and females. This distinction is thought to potentially reflect sexual size dimorphism that may drive prey shifts (altering diet) as well as metabolic differences that are not evident in the other marine mammal species studied from this perspective (Nelson et al., 2013b). Similar evaluations of cetacean epidermal microbiota include one study reporting no significant differences between sexes in microbiota structure of Humpback Whales (*Megaptera novaeangliae* (Borowski, 1781)) (Apprill et al., 2014), but another finding significant sex differences in Beluga whales (*Delphinapterus leucas* (Pallas, 1776)) (Van Cise et al., 2020). The latter is thought to potentially be driven by either endogenous differences (e.g. hormone levels, group associations, dietary differences) or sex-specific habitat preference (e.g. males and females may differ in preferences for shore proximity and ice concentration) (Hauser et al., 2017; Van Cise et al., 2020).

Fish represent the most diverse vertebrate Class, with an estimated 35,934 described species compared to the next most speciose Class, Reptilia (estimated 11,570 species) (IUCN, 2021). The high species diversity of fish makes this clade especially important for studying the microbiota, and in particular, determining how host-microbe associations might impact evolutionary trajectories that shape biodiversity. Fish are also of high importance both in terms of the global economy and food security (Food and Agriculture Organisation of the United Nations [FAO], 2016), with improvements in aquaculture benefiting both. Studies to date have demonstrated an important link between the gut microbiota and fish sex, with males and females differing in both alpha and beta diversity (Li et al., 2016; DeBofsky et al., 2020).

In addition to the evidence for innate sexual differences in the microbiota of fish, several studies have demonstrated sex-specific microbial responses to environmental factors. In zebrafish (*Danio rerio* (Hamilton, 1822)), exposure to titanium dioxide in combination with bisphenol A shifted the gut microbiota, neurotransmission, epithelial permeability, inflammation, and oxidative stress in a sex-specific manner (Chen et al., 2018a). Similarly, in fathead minnows (*Pimephales promelas* Rafinesque, 1820), females exhibited higher gut bacterial alpha diversity, differences in beta diversity, taxa abundance, and predicted functional pathways relative to males (DeBofsky et al., 2020). The gut microbiota of males and females of this species also responded differently to low-dose exposure of the polycyclic hydrocarbon BaP (PAH benzo[a]pyrene), with exposure disturbing the gut microbial community structure of females but not males (DeBofsky et al., 2020). In three-spined stickleback (*Gasterosteus aculeatus* Linnaeus, 1758) and Eurasian perch (*Perca fluviatilis* Linnaeus, 1758), among-individual diet variation was correlated with individual differences in gut microbiota in a sex-dependent fashion, a result further confirmed by experimental dietary manipulation (Bolnick et al., 2014). In another study of Eurasian perch, elements of the gut microbial community were found to react to predation stress and food rationing in a sex-dependent manner (Zha et al., 2018). The sex-dependent response of the microbiota to environmental changes observed across fish taxa (and other vertebrate classes) poses important questions in terms of our approach to microbial manipulation to manage host health, demonstrating the importance of considering the effect of sex in any such intervention.

## Case Study: Studying the effect of sex on the microbiota using *Potamopyrgus antipodarum*

Some animal taxa exist in both sexual and asexual forms (e.g. Neiman et al., 2014), providing a powerful model to examine microbial differences between males, females, and asexual individuals while controlling for host lineage. One such species is *Potamopyrgus antipodarum* (Gray, 1843)—an aquatic snail native to New Zealand freshwater lakes and streams (Winterbourn, 1973). *P. antipodarum* is characterized by the existence of multiple triploid and tetraploid asexual lineages that are separately derived from diploid sexual conspecifics (Lively, 1987; Neiman et al., 2011). This reproductive mode and ploidy variation, combined with the ability to easily collect from the field and maintain and culture in the laboratory, has led to these snails achieving prominence as a model system for the evolution of sex (Lively, 1987; Neiman et al., 2011). These same strengths are now being leveraged in microbiota research, where *P. antipodarum* is being used to assess the impact of reproductive mode in shaping host microbial communities.

Recent studies have shown that snail microbiota composition varies substantially among native New Zealand populations (Takacs-Vesbach et al., 2016), between native and invasive populations (Bankers et al., 2021), and between sexual and asexual forms (Takacs-Vesbach et al., 2016; Bankers et al., 2021). Although ploidy is a confounding factor in the latter comparison, the variance between the bacterial communities of sexual vs. asexual populations (representing multiple lakes) was more than two times greater than between those of triploid and tetraploid populations. While these data hint that reproductive mode is a more important factor than ploidy in determining *P. antipodarum* microbiota, ploidy is nevertheless worth exploring more broadly. Triploid and tetraploid *P. antipodarum* did tend to harbour different microbial communities (Takacs-Vesbach et al., 2016), and ploidy level can influence immune function and host resistance (King et al., 2012). For this latter reason, the role of ploidy in host control of the microbiota (Foster et al., 2017) is an especially interesting avenue going forward. With few exceptions (e.g. Cavé-Radet et al., 2019; Forrester and Ashman, 2018), links between animal ploidy level and microbial community composition are unclear and may shed light on how host biological differences can drive microbiota variation.

Specific bacterial taxa also differ in their prevalence between sexual and asexual *P. antipodarum* across New Zealand lakes (Takacs-Vesbach et al., 2016; Bankers et al., 2021). Perhaps, the most intriguing difference was reported by Takacs-Vesbach et al. (2016), who found that Rickettsiales were absent in asexual snails but present in sexual males and females, regardless of lake origin. Members of Rickettsiales have a wide host range and operate across the parasite-mutualist continuum (Perlman et al., 2006), with some members driving sex ratio distortion (Lawson et al., 2001; von der Schulenburg et al., 2001). Colonization of Rickettsiales in male and female sexual snails from both field and lab cultures suggests that these symbionts might be inherited (Takacs-Vesbach et al., 2016). Conversely, asexual snails across field populations and lab cultured lineages were enriched for bacteria closely related to the Proteobacteria genus *Rhodobacter*. Members of the *Rhodobacter* genus are phototrophic in aquatic environments and have been found to be symbionts of marine sponges (Althoff et al., 1998) and *Daphnia* (Qi et al., 2009). That *Rhodobacter* was found in both adults and juveniles from one lake suggests that this bacterium might also be inherited and potentially of functional importance in asexual animals.

A more recent study found that even within the same New Zealand lake, *P. antipodarum* microbial community structure differed by reproductive strategy and sex. Ten amplicon sequence variants (ASVs) (all Xanthomonadaceae) were significantly more abundant in asexuals than sexuals (Bankers et al., 2021), and fifty ASVs (over-represented by *Niabella*, *Bacillus*, and OM60) were significantly more abundant in male than female snails. Overall, the differences in microbiota structure identified between male and female or sexual and asexual *P. antipodarum* demonstrate how host sexual systems can greatly influence the microbiota. The relevance of these findings will be enhanced through future work examining whether sexual differences in the microbiota of *P. antipodarum* are of functional significance to host health or host evolutionary trajectories.

## A role of the microbiota in sex differentiation?

The broad diversity of sex-determining systems makes aquatic animals especially good models for research. Simultaneous or sequential hermaphroditism occurs across a range of aquatic invertebrates including sponges, arthropods, echinoderms, and molluscs, while among vertebrates occurs only in fish (Policansky, 1982). Animals that are simultaneous hermaphrodites have both male and female gonads, while sequential hermaphrodites change from one sex to another within an individual’s lifetime (Warner, 1975; Munday et al., 2006). Sequential hermaphroditism is broadly classified into three categories based on the modality of transition: (1) female to male (protogynous), (2) male to female (protandrous), or (3) serial sex change (bi-directional). In sequential hermaphroditism, sex change is typically driven by body size, age, or community social structure and results in changes in reproductive behaviour, gonadal anatomy, and external morphology (Warner, 1975; Munday et al., 2006; Godwin, 2009; Todd et al., 2016; Liu et al., 2017).

As the only vertebrate group known to exhibit sequential hermaphroditism, teleost fish offer a unique insight into the biological basis of sex. Mechanistically, sex change in teleost fish appears to be governed by a complex interplay of factors including host neurology, hormone balance, stress pathways, and epigenetics (reviewed in Gemmell et al., 2019; Todd et al., 2016). The Hypothalamic–Pituitary–Gonadal (HPG) and Hypothalamic–Pituitary–Interrenal (HPI) axes are involved in oestrogen/androgen balance and release of stress hormones (glucocorticoid steroids), respectively, and are considered the major neuroendocrine system components underpinning sex change in fish (Todd et al., 2016; Goikoetxea et al., 2017; Liu et al., 2017). Of particular importance are 11-ketotestosterone (11-KT) and 17β-estradiol (E2), which respectively promote testicular and ovarian function (Devlin & Nagahama, 2002; Godwin, 2010; Todd et al., 2016; Gemmell et al., 2019). When levels of 11-KT and E2 are altered experimentally, the result is promotion of masculinization or feminization in fish (Chang et al., 1995; Higa et al., 2003; Yeh et al., 2003).

There is some evidence that the microbiota is important in modulating the HPG/HPI axes in fish (Avella et al., 2012; Davis et al., 2016) and could be a missing link in our understanding of sequential hermaphroditism and sex determination. Some of the most convincing evidence for a microbial role in sex determination comes from studies of zebrafish (*Danio rerio*). *D. rerio* is a juvenile protogynous hermaphrodite species (Takahashi, 1977) that first develops ovary-like gonads before some individuals undergo bisexual differentiation, whereby ovaries enter an intermediate phase termed “altered ovary” before finally forming testes (Maack & Segner, 2003). Chronic administration of the probiotic *Lactobacillus rhamnosus* to juvenile *D. rerio* from the time of first feeding up until 9 weeks post-fertilization has been found to result in 93% females and 7% males in the control group, compared to 55% females and 45% males in the probiotic group (Avella et al., 2012). This study also reported increased expression of gonadotropin-releasing hormone 3 (GnRH3), which is thought to elicit gonadotropin release (which acts as an upstream regulator of sex steroids) (Kuo et al., 2005) and sexual differentiation in this species (Abraham et al., 2009). A possible mechanistic basis for this finding has been deduced by demonstrating that *L. rhamnosus* activates the HPG axis of *D. rerio* via increased production of the hormone leptin, which in turn is correlated with a rise in brain gene expression of *kiss1* and *kiss2* and an increase in GnRH3 expression (Gioacchini et al., 2010). Activation of the HPG axis and GnRH transcription critically depends on adequate host energy stores (Hill et al., 2008), the magnitude of which are signalled to the hypothalamus by neuropeptide hormones and metabolic signals such as *kiss1*, *kiss2*, and leptin (Fernandez-Fernandez et al., 2006; Castellano et al., 2009; Kitahashi et al., 2009) that subsequently moderate GnRH expression (Smith et al., 2002; Barb et al., 2005). The link between host energy stores, leptin, *kiss1*, *kiss2*, and GnRH expression may, therefore, be applicable more broadly across sequentially hermaphroditic fish, where sex change may be dependent on host size (Ross et al., 1983).

The action of the products of microbial metabolism such as short-chain fatty acids (SCFAs) has also been shown to impact the HPG axis in other fish taxa. For example, dietary modification by addition of the SCFA butyrate reversed the androgenic effects of a plant-based diet in gilthead sea bream (*Sparus aurata* Linnaeus, 1758) (Simó-Mirabet et al., 2018), while another study demonstrated changes in the microbiota associated with age and sex in this species (Piazzon et al., 2019). These findings warrant further investigation, indicating a possible mechanistic link between host environmental cues (in this case diet), microbial metabolism, and subsequent host hormonal changes that may initiate or contribute to the sex change process.

There is a relatively large body of research on the interaction between the microbiota, the HPG axis, and sexual phenotypes in other vertebrates. This work could also be of broader relevance to fish and other sequentially hermaphroditic animals. For example, the level of Gonadotropin Releasing Hormone (GnRH), which stimulates release of Leuteinizing Hormone (LH) and Follicle-stimulating Hormone (FSH), can be impacted by the presence of certain microbes in both birds and mammals (Wang et al., 2017; Haziak et al., 2018; Lee et al., 2019). Similarly, the gut microbiota has been directly linked with circulating levels of gonadotropins (LH and FSH) and sex steroids (testosterone, oestrogen, progesterone) across several mammalian taxa (Markle et al., 2013; Al-Asmakh et al., 2014; Poutahidis et al., 2014; Lindheim et al., 2017; Shin et al., 2019; Xu et al., 2020). Of particular relevance are experimental mammalian studies that have demonstrated that gut microbiota influence both sex hormone levels and other host phenotypes. For example, a study in mice found that microbiota transplantation from males to females increased circulating testosterone to a sufficient degree to modify autoimmune disease risk (Markle et al., 2013). Moreover, male mice supplemented with *Lactobacillus reuteri* have been shown to have increased circulating testosterone and testicular weight relative to controls (Poutahidis et al., 2014). Similar results have been demonstrated in a study of germ-free mice colonized with a microbial community (Al-Asmakh et al., 2014).

## Microbial sex distortion in aquatic animals

In extreme cases, microbial symbionts have evolved mechanisms to skew the sex ratio of host populations to favour their own fitness (Hurst & Frost, 2015). This phenomenon is particularly prevalent among arthropods, with approximately 52% of aquatic insects estimated to carry the feminizing bacterial symbiont *Wolbachia* (Sazama et al., 2017). Microbe-driven sex distortion has also been reported in aquatic crustaceans (Bouchon et al., 1998; Terry et al., 1998; Ironside et al., 2003). For example, in the aquatic isopod *Gammarus deubeni* Lilljeborg, 1852, two species of eukaryotic microbes belonging to the Microspora phylum have been shown to drive host feminization by inhibiting development of the androgenic gland (Jahnke et al., 2013). Sex-distorting symbionts have been proposed to be important drivers of the evolution of sex-determination systems (Cordaux et al., 2011), with evidence from terrestrial isopods (the common pillbug *Armadillidium vulgare* (Latreille, 1804)) that horizontal gene transfer from the endosymbiont *Wolbachia* drives the evolution of novel sex chromosomes (Leclercq et al., 2016). Examining sex-distorting symbionts in aquatic hosts, with their diversity of sex-determining mechanisms, could yield new discoveries and advance our understanding of evolutionary transitions in animal sex-determining systems.

## Importance in aquaculture and conservation

Aquatic ecosystems are of great importance for food security, the economy, and biodiversity (Geist, 2011; Food and Agriculture Organisation of the United Nations [FAO], 2016). While the microbiota has been shown to be important in growth rate (Lopez Cazorla et al., 2015; Zuo et al., 2019) and disease susceptibility (Boutin et al., 2013; Sha et al., 2016) in aquatic animals, the overall factors governing microbiota structure (including host sex) are poorly understood.

The uses of probiotics (microbes conferring health benefits to their hosts) and prebiotics (substrates utilized by microbes that confer host health benefits) (Sanders et al., 2019) have become a key focus in aquaculture and aquatic species conservation (Robertson et al., 2000; Refstie et al., 2010; Dias et al., 2012; Akhter et al., 2015; Hai, 2015; Mehrim et al., 2015; Ringø, 2020). Despite the recognition that the effects of pre/probiotics can vary between sexes in mammals (Shastri et al., 2015; Christoforidou et al., 2019), few studies have taken sex into account in aquaculture research (Mehrim et al., 2015). Addressing this oversight is critically important given that optimal host responses to probiotic treatment can differ between sexes (Mehrim et al., 2015) and that accounting for sexual differences in species is important for successful species conservation outcomes (Gantchoff et al., 2019).

## Conclusion and future directions

As studies examining the importance of host sex and the microbiota garner increasing attention in the terrestrial realm, our understanding of this topic in aquatic hosts remains limited. We emphasize that sexual differences in the microbiota exist across diverse aquatic animal hosts yet much remains unknown in terms of what drives these differences and why sexual differences in the microbiota occur in some species but not others.

Crucially, extending studies on microbe associations across sexes in aquatic animals offers much more than simply “filling in the gaps” in our understanding of host-associated microbial diversity. That many aquatic animals have such diverse sex-determining mechanisms and sexual plasticity, makes these organisms powerful models to examine how the microbiota interacts with, or even shapes host sex. Although experimental work has demonstrated a role of microbes in sequential hermaphroditism in fish, much more research is needed encompassing wider host taxa.

A key question is how often microbes play a role in initiating the sex change process, which in itself will require more advanced studies on microbial sex distortion, as well as the gut-brain axis and neuroendocrine pathways of teleosts and other sequentially hermaphroditic taxa. Ultimately, this avenue of research will be valuable in our understanding of the intersection of biological sex determination and microbiology and more broadly yield key models to advance our knowledge of vertebrate development (including in humans), particularly in terms of normal and atypical sexual development.

Future studies investigating the microbiota and aquatic animal health should also include sex as a variable (where appropriate). The information regarding sexual differences in the response to stressors or in the efficacy of pre/probiotic therapies will undoubtedly have potentially long-term pay-offs in terms of managing aquatic animal health.

## Data Availability

Data used to generate Fig. 1 are available upon request from the authors.
